# Minimally Invasive Management of Gastric Outlet Obstruction Secondary to Cholecystogastric Fistula With Gallstone Impaction: A Case Report

**DOI:** 10.7759/cureus.83250

**Published:** 2025-04-30

**Authors:** Jacob Sapell, Mustafa Al-Roubaie, Elias Salloum, Hakob Kocharyan

**Affiliations:** 1 Radiology, University of South Florida (USF) Health Morsani College of Medicine, Tampa, USA; 2 Interventional Radiology, Moffitt Cancer Center, Tampa, USA

**Keywords:** biliary fistula, cholecystogastric fistula, endoscopic lithotripsy, gallstone impaction, gastric outlet obstruction, gastrojejunostomy, spyglass

## Abstract

Cholecystogastric fistulas are rare complications of gallstone disease that can lead to significant clinical scenarios, one of which is gastric outlet obstruction. This case underscores the utility of minimally invasive techniques in managing biliary conditions.

We present a case of a 63-year-old female Jehovah’s Witness with a significant medical history, including iron and B12 deficiency anemia, chronic gallstone disease, and squamous cell carcinoma of the hypopharynx, who presented with severe abdominal pain and nausea. Diagnostic imaging revealed a cholecystogastric fistula with a large gallstone causing gastric outlet obstruction. The patient was deemed a non-surgical candidate. Conventional endoscopy was not an option either, given the history of a high-grade hypopharyngeal stricture from prior cancer treatment. Multiple minimally invasive procedures were performed, including the conversion of a gastrostomy tube (G-tube) to a gastrojejunostomy tube (GJ-tube), and percutaneous endoscopic lithotripsy using the SpyGlass system (Boston Scientific Corporation, Marlborough, MA, USA).

The initial conversion of the G-tube to a GJ-tube improved feeding tolerance and provided symptomatic relief. Endoscopic lithotripsy successfully fragmented the gallstone, although complete removal was not achieved in the first session. The patient’s condition improved, with reduced abdominal pain and better nutritional management, and further follow-up procedures were planned to address the residual stone.

This case illustrates the effectiveness of minimally invasive techniques, such as endoscopic lithotripsy, in managing complex biliary conditions. These approaches are especially critical for patients who are poor surgical candidates or refuse blood products, as surgical intervention poses heightened risks in managing potential hemorrhagic complications.

## Introduction

Cholecystoenteric fistulas are typically caused by large or chronically impacted gallstones eroding through the gallbladder wall into adjacent gastrointestinal structures. These fistulas occur in approximately 0.3%-5% of patients with cholelithiasis [1]. Of these, cholecystogastric fistulas comprise 5%-10% of cases, resulting from chronic inflammation and erosion of the gallbladder wall into the stomach [2].

Cholecystogastric fistulas can lead to complex clinical scenarios, most notably recurrent cholecystitis and gastric outlet obstruction due to impacted gallstones [3]. Given these potential complications, management often involves a combination of surgical and endoscopic techniques.

Minimally invasive procedures, such as endoscopic lithotripsy, have become essential in managing complex biliary and pancreatic ductal stones, and can be utilized in select cases of gastric outlet obstruction. Endoscopic lithotripsy techniques include mechanical lithotripsy, electrohydraulic lithotripsy (EHL), laser lithotripsy, and extracorporeal shockwave lithotripsy (ESWL). These approaches aim to fragment large or impacted stones under direct visualization, reducing the need for more invasive surgical procedures. The SpyGlass system (Boston Scientific Corporation, Marlborough, MA, USA) provides direct visualization and enables targeted stone fragmentation, making it a valuable tool in high-risk surgical candidates [4].

## Case presentation

We present the case of a 63-year-old female Jehovah’s Witness with a medical history significant for iron and B12 deficiency anemia secondary to chronic malnutrition, and squamous cell carcinoma of the hypopharynx. She underwent chemoradiotherapy four years prior, which resulted in complete stenosis of the cervical esophagus and subsequent placement of a gastrostomy tube (G-tube) for enteral feeding. The patient had passed a gallstone per rectum approximately one year prior to presentation. She sought medical attention due to severe abdominal pain and nausea that had persisted for two days, following a recent G-tube replacement three days prior to presentation.

Upon examination, the patient described experiencing deep, burning pain and nausea, with an urge to vomit, but was unable to do so due to the stenosis of the hypopharynx. She reported that venting the G-tube provided temporary relief from the abdominal pain and drained the contents she had ingested for feeding, which included dark, coffee-ground colored material.

**Figure 1:**
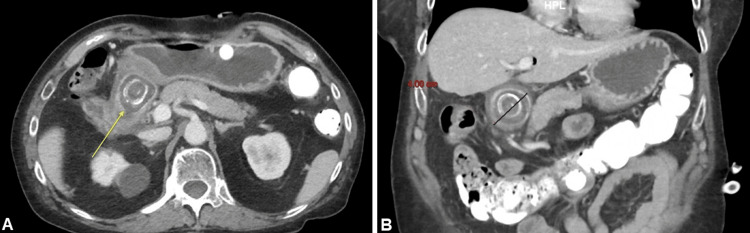
(A) Yellow arrow pointing to the initial axial CT of the abdomen, showing a large 4 cm gallstone impacted in the gastric outlet. (B) Initial coronal CT of the abdomen showing the same gallstone impacting the gastric outlet.

Laboratory tests revealed a declining hemoglobin level over four days, from 14.0 g/dL to 9.6 g/dL. This decline was likely due to an acute gastrointestinal bleed, which could be secondary to the fistulous connection and/or recent G-tube replacement, though other factors may also have contributed. A CT scan of the abdomen and pelvis showed a contracted gallbladder with thickened walls and pericholecystic fluid, suggesting cholecystitis with a fistulous connection to the pyloric channel or proximal duodenum. A large gallstone, measuring approximately 4 cm, was identified in the pyloric channel, causing inflammatory changes (Figure 1).

**Figure 2:**
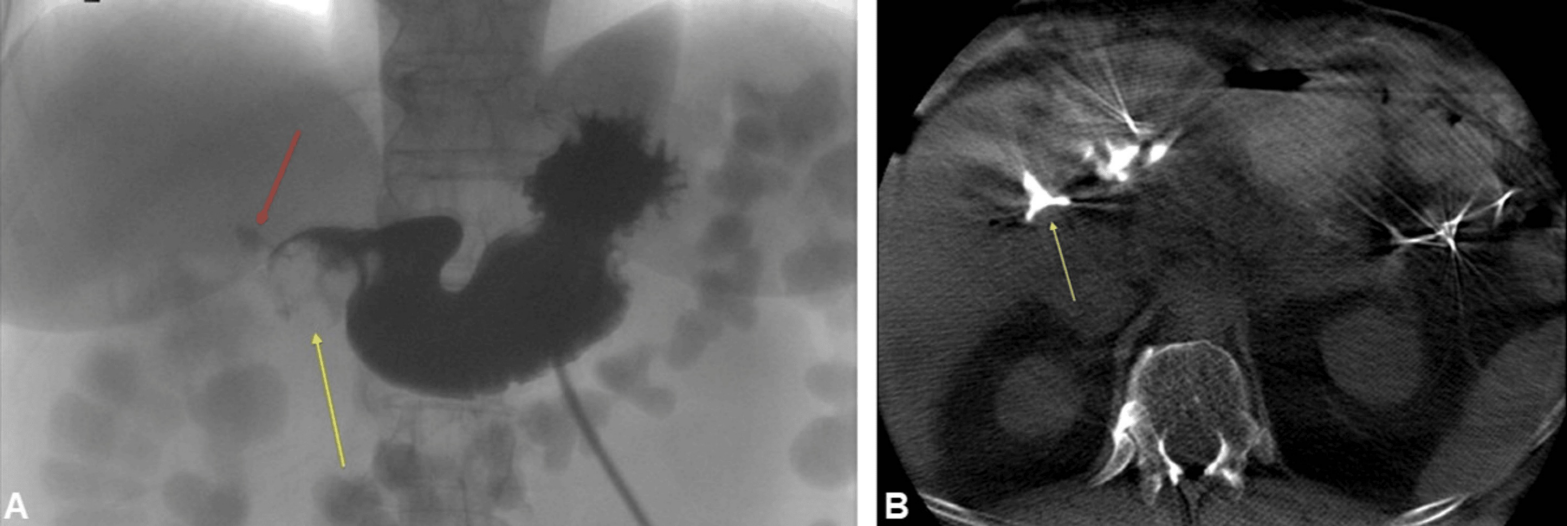
(A) Upper GI study showing a radiolucent round structure (yellow arrow) in the pylorus/first portion of the duodenum. The red arrow points to contrast extension to the gallbladder fossa in the context of a cholecystogastric/duodenal fistula. (B) Initial cone beam CT enterography with contrast injection through the gastrostomy tube, demonstrating contrast extension to the contracted gallbladder, indicating fistulous communication (yellow arrow).

**Figure 3:**
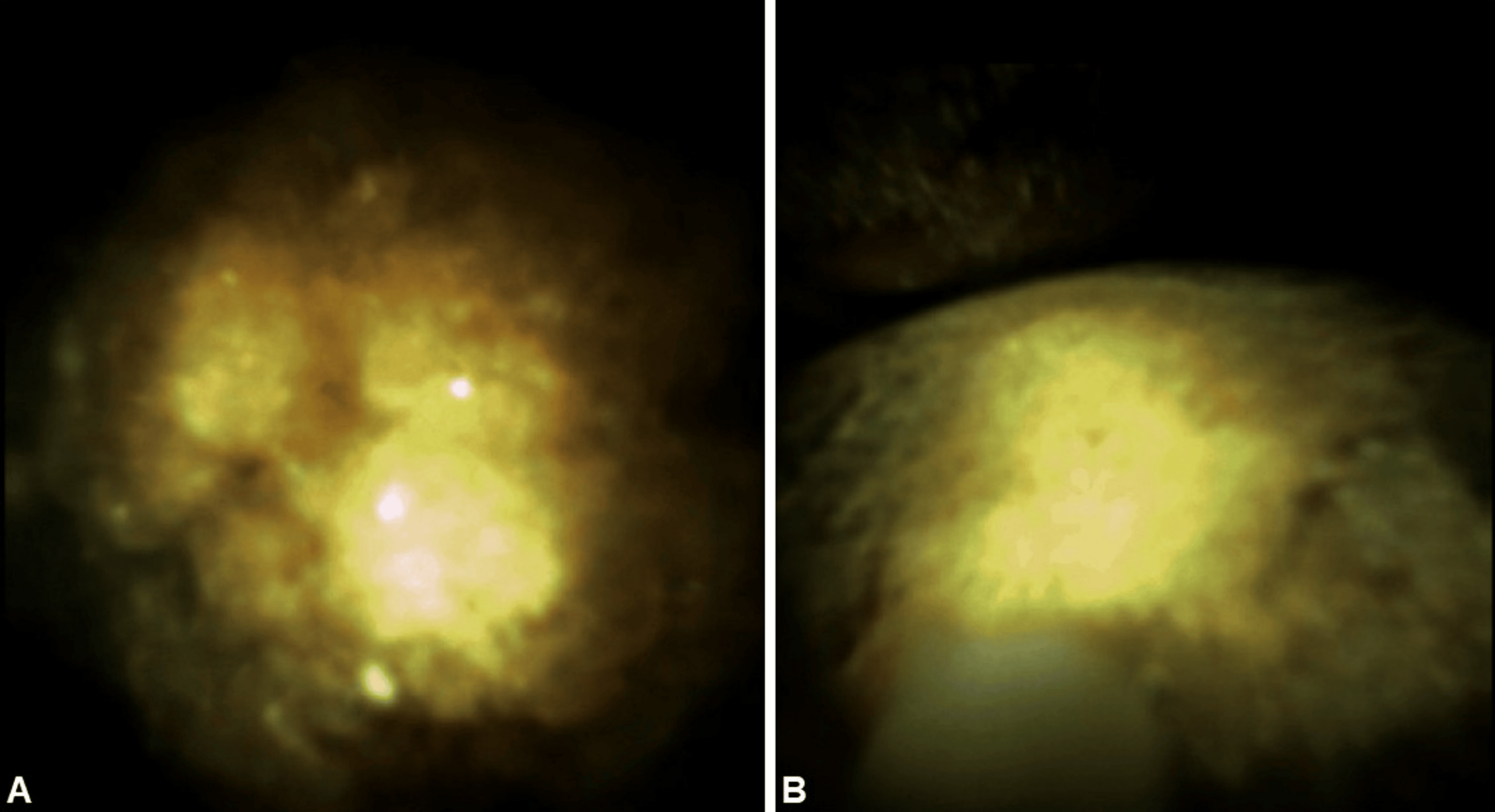
(A-B) Endoscopic pictures of the large gallstone impacting the gastric outlet.

**Figure 4:**
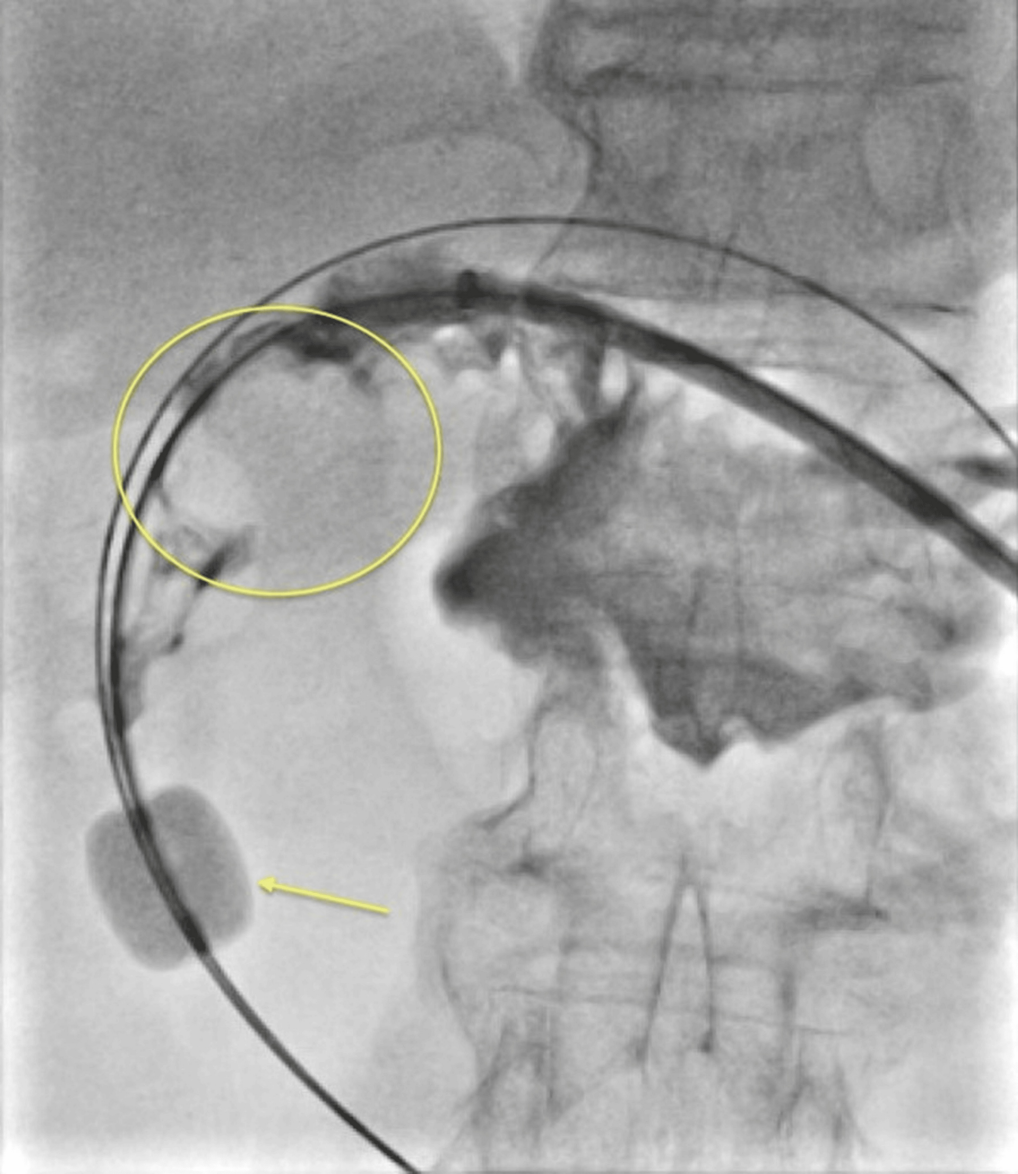
Initial enterography with a yellow circle showing a large radiolucency corresponding to the impacted gallstone, and a Fogarty balloon (yellow arrow) inflated distally to aid disimpaction of the stone via retrieving maneuver.

**Figure 5:**
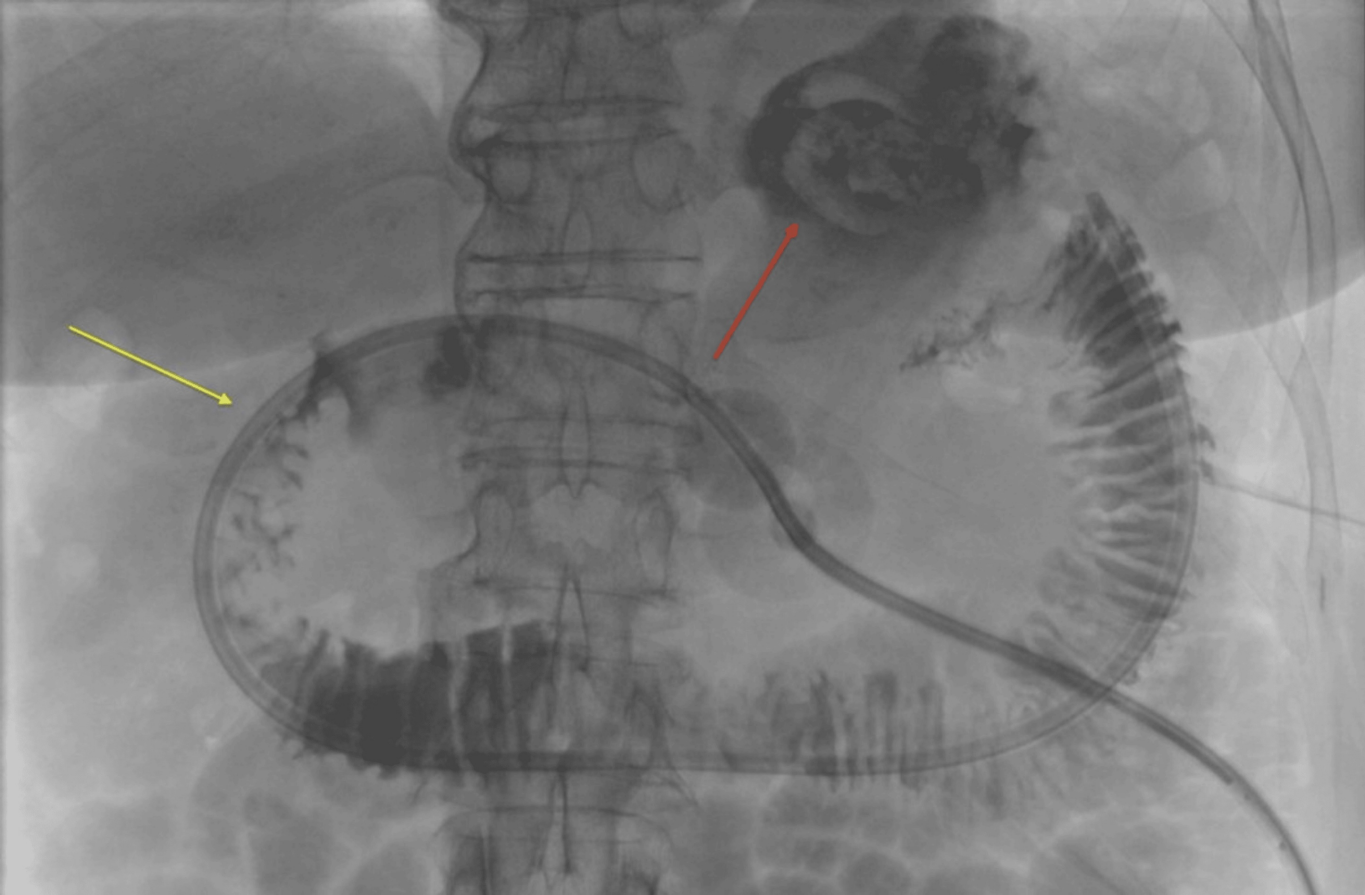
Enterography through the gastrojejunostomy tube: gallstone dislodgement to the gastric fundus (red arrow), and restoration of the gastric outlet (yellow arrow).

The gastroduodenoscopy revealed large gallstones that had eroded into the pyloric channel, consistent with a cholecystogastric fistula and high-grade gastric outlet obstruction (Figures 2-3). A percutaneous gastroduodenoscopy was conducted via the existing gastrostomy tract. A balloon was inflated distal to the stone with retracting maneuvers; however, no appreciable disimpaction was achieved (Figure 4). The G-tube was converted to a gastrojejunostomy tube (GJ-tube) to improve feeding tolerance and reduce inflammatory changes associated with post-pyloric feeding (Figure 5).

**Figure 6:**
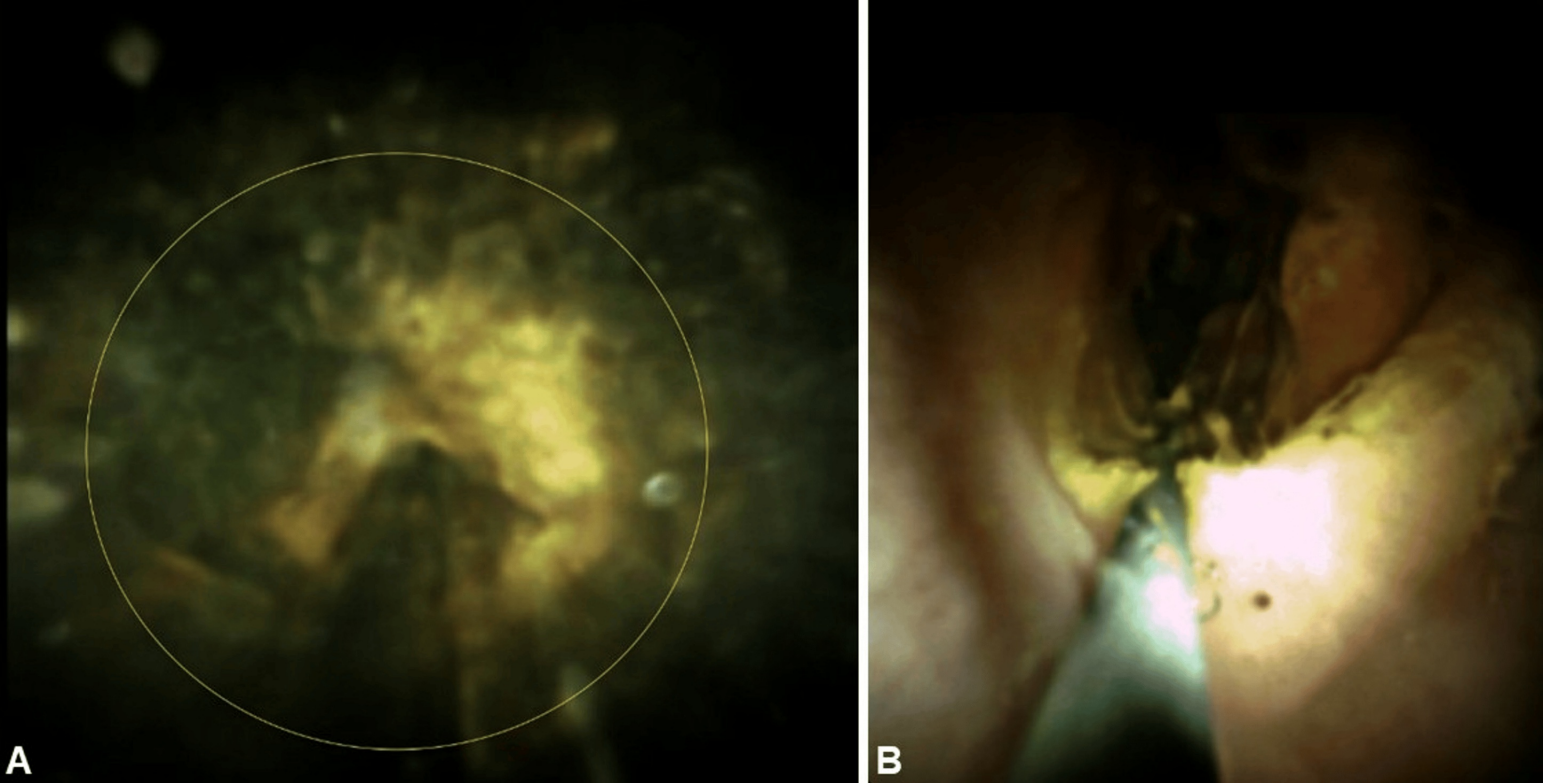
(A) Yellow circle pointing to the tip of the lithotripsy wire and the indentation made on the stone surface during the session. (B) Final percutaneous gastroduodenoscopy demonstrating the passage of the stone and restoration of the gastric outlet.

**Figure 7:**
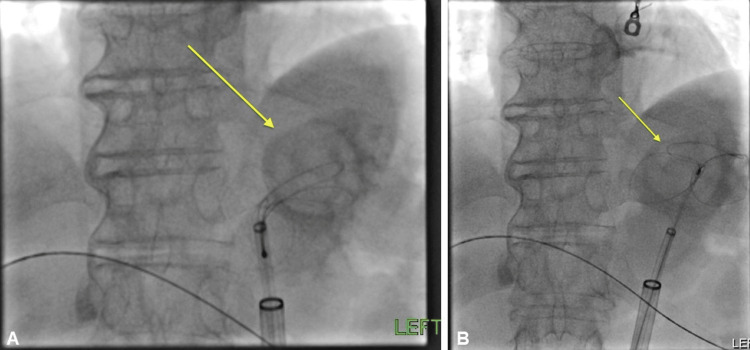
(A-B) Attempted snare extraction of the gallstone from the gastric fundus (yellow arrows).

Five weeks later, endoscopic lithotripsy using the SpyGlass system was performed to attempt to fragment the large gallstone. The stone, at this time, was found freely floating in the gastric lumen. Two lithotripsy wires were used, with the delivery of 3,400 pulses. As a result, several fragments broke off the dominant stone (Figure 6A). Extraction was attempted again through the (dilated) gastrostomy tract but was not possible due to the sizable dimension of the stone remnant (Figure 7). A Kelly clamp was used to try to break it down, and several “scoops” of stone content were removed, but the stone migrated back to the gastric cardia. It was decided to replace the GJ-tube and address further stone removal in subsequent sessions of lithotripsy.

**Figure 8:**
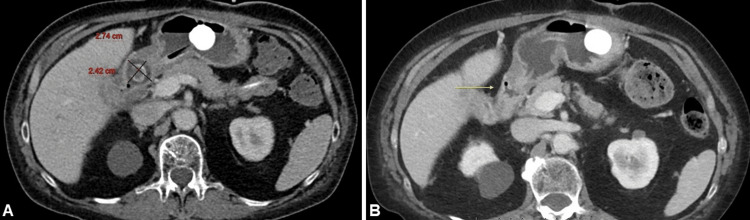
(A) Axial plane CT of the upper abdomen after lithotripsy, showing significant reduction in the gallstone size. (B) Final CT of the abdomen demonstrates passage of the stone and marked reduction in the periduodenal inflammatory changes (yellow arrow).

A subsequent CT demonstrated a residual smaller stone in the pyloric channel (Figure 8A). During the next GJ-tube replacement, endoscopy demonstrated a mildly stenotic first segment of the duodenum, some chronic inflammatory changes, and the stone was not visualized (Figure 6B).

The patient was advised to start a trial of gastric feeds, which she tolerated well. A short-term repeat CT demonstrated passage of the stone (Figure 8B). The GJ-tube was eventually converted back to a G-tube.

Throughout her treatment, the patient’s condition was managed by minimally invasive methods. She was closely monitored for signs of recurrent bleeding, and her nutritional needs were managed via the GJ-tube. The patient remained stable, with improved feeding tolerance and reduced abdominal pain, as she continued to undergo periodic evaluations and follow-up procedures to address the residual gallstone and ensure the patency of the GJ-tube.

## Discussion

Cholecystogastric fistulas with gallstone migration leading to gastric outlet obstruction are rare complications of gallstone disease, often presenting as Bouveret's syndrome [5,6]. Endoscopic intervention is generally considered first-line treatment; however, success rates vary significantly depending on stone size and location. For larger stones (>2.5-3 cm), endoscopic retrieval success rates drop considerably, with reports suggesting rates no higher than 58% [7].

In our case, the presence of a 4 cm stone posed substantial technical challenges. While endoscopic baskets or snares can effectively retrieve smaller stones, larger or impacted stones frequently necessitate lithotripsy techniques, such as EHL, laser lithotripsy, or ESWL [5,8]. Although precise success rates for endoscopic lithotripsy in Bouveret’s syndrome are difficult to define due to the rarity of the condition and variability in techniques, among reported cases of successful endoscopic management, mechanical lithotripsy and EHL have been the most frequently utilized approaches, accounting for 40% and 21% of cases, respectively, while laser lithotripsy was employed in 15% [7]. Despite these advancements, complete stone clearance often requires multiple sessions, and success remains limited in cases involving large stones or altered anatomy.

The adaptation of the SpyGlass system, which is primarily designed for biliary interventions, allowed for direct visualization and targeted fragmentation in this gastrointestinal setting. This approach aligns with evolving strategies, where cholangioscopy-guided lithotripsy is employed for complex gastric outlet obstructions when conventional endoscopy or surgery is not feasible [8]. The advantages of the SpyGlass system include enhanced visualization and precise lithotripsy delivery in a minimally invasive manner. However, its limitations are evident in cases involving large or heavily calcified stones, where multiple sessions may be required, and complete clearance is not always achievable in a single procedure [7,8]. In our patient, a staged minimally invasive strategy was employed. This began with GJ-tube placement to bypass the obstruction and maintain nutrition, followed by SpyGlass-assisted lithotripsy, which successfully relieved symptoms without requiring surgical intervention.

Surgical management, including enterolithotomy with fistula repair and cholecystectomy, remains the definitive treatment but carries significant risk, particularly in patients with comorbidities or contraindications to surgery [5,6]. In our case, the patient's refusal of blood products further limited surgical options, reinforcing the value of a minimally invasive approach. Similar to previously published cases, our management prioritized endoscopic techniques due to high surgical risk, with partial stone fragmentation providing effective symptomatic relief. This reflects reported strategies, where even incomplete lithotripsy can effectively manage obstruction in patients unsuitable for definitive surgical repair [5,6].

This case highlights the role of individualized management in addressing complex gallstone-related obstructions. It also demonstrates how biliary tools, like SpyGlass, can be effectively repurposed for gastrointestinal applications in high-risk patients.

## Conclusions

Despite the success of endoscopic lithotripsy in this case, the long-term efficacy of percutaneous and endoscopic approaches for gallstone-related gastric outlet obstruction remains uncertain. Further studies are needed to assess recurrence risk, evaluate patient selection criteria, and optimize procedural techniques to enhance stone clearance. Additionally, comparing endoscopic lithotripsy to surgical management in high-risk patients would provide valuable data on long-term outcomes and complication rates.

This case illustrates the efficacy of minimally invasive techniques such as percutaneous endoscopic lithotripsy in managing complex biliary stone fistulas, particularly in cases where conventional endoscopy is not feasible.
